# A Simple Standard for Sharing Ontological Mappings (SSSOM)

**DOI:** 10.1093/database/baac035

**Published:** 2022-05-25

**Authors:** Nicolas Matentzoglu, James P Balhoff, Susan M Bello, Chris Bizon, Matthew Brush, Tiffany J Callahan, Christopher G Chute, William D Duncan, Chris T Evelo, Davera Gabriel, John Graybeal, Alasdair Gray, Benjamin M Gyori, Melissa Haendel, Henriette Harmse, Nomi L Harris, Ian Harrow, Harshad B Hegde, Amelia L Hoyt, Charles T Hoyt, Dazhi Jiao, Ernesto Jiménez-Ruiz, Simon Jupp, Hyeongsik Kim, Sebastian Koehler, Thomas Liener, Qinqin Long, James Malone, James A McLaughlin, Julie A McMurry, Sierra Moxon, Monica C Munoz-Torres, David Osumi-Sutherland, James A Overton, Bjoern Peters, Tim Putman, Núria Queralt-Rosinach, Kent Shefchek, Harold Solbrig, Anne Thessen, Tania Tudorache, Nicole Vasilevsky, Alex H Wagner, Christopher J Mungall

**Affiliations:** Semanticly Ltd, London WC2H 9JQ, UK; RENCI, University of North Carolina, Chapel Hill, NC 27517, USA; The Jackson Laboratory, Bar Harbor, ME 04609, USA; RENCI, University of North Carolina, Chapel Hill, NC 27517, USA; University of Colorado Anschutz Medical Campus, Aurora, CO 80217, USA; University of Colorado Anschutz Medical Campus, Aurora, CO 80217, USA; Johns Hopkins University, Baltimore, MD 21210, USA; Lawrence Berkeley National Laboratory, Berkeley, CA 94720, USA; Maastricht University, Maastricht 6211 LK, The Netherlands; Johns Hopkins University, Baltimore, MD 21210, USA; Stanford University, Stanford, CA 94305, USA; Department of Computer Science, Heriot-Watt University, Edinburgh, Currie EH14 4AS, UK; Harvard Medical School, Boston, MA 02115, USA; University of Colorado Anschutz Medical Campus, Aurora, CO 80217, USA; European Bioinformatics Institute (EMBL-EBI), Hinxton CB10 1SD, UK; Lawrence Berkeley National Laboratory, Berkeley, CA 94720, USA; Pistoia Alliance Inc, USA; Lawrence Berkeley National Laboratory, Berkeley, CA 94720, USA; Beth Israel Deaconess Medical Center, Boston, MA 02215, USA; Harvard Medical School, Boston, MA 02115, USA; Johns Hopkins University, Baltimore, MD 21210, USA; City University of London, London EC1V 0HB, UK; University of Oslo, Oslo 0315, Norway; SciBite Limited, Bio Data Innovation Centre, Wellcome Genome Campus, Hinxton, Saffron Walden CB10 1DR, UK; Robert Bosch LLC, Sunnyvale, CA 94085, USA; Ada Health GmbH, Berlin 10178, Germany; Pistoia Alliance Inc, USA; Leiden University Medical Center, Leiden 2333 ZA, The Netherlands; BenchSci, 25 York St Suite 1100, Toronto, ON M5J 2V5, Canada; European Bioinformatics Institute (EMBL-EBI), Hinxton CB10 1SD, UK; University of Colorado Anschutz Medical Campus, Aurora, CO 80217, USA; Lawrence Berkeley National Laboratory, Berkeley, CA 94720, USA; University of Colorado Anschutz Medical Campus, Aurora, CO 80217, USA; European Bioinformatics Institute (EMBL-EBI), Hinxton CB10 1SD, UK; Knocean Inc., Toronto, ON M6P 2T3, Canada; La Jolla Institute for Immunology, 9420 Athena Circle, La Jolla, CA 92037, USA; University of Colorado Anschutz Medical Campus, Aurora, CO 80217, USA; Leiden University Medical Center, Leiden 2333 ZA, The Netherlands; University of Colorado Anschutz Medical Campus, Aurora, CO 80217, USA; Johns Hopkins University, Baltimore, MD 21210, USA; University of Colorado Anschutz Medical Campus, Aurora, CO 80217, USA; Independent Scholar; University of Colorado Anschutz Medical Campus, Aurora, CO 80217, USA; The Steve and Cindy Rasmussen Institute for Genomic Medicine, Nationwide Children’s Hospital, Columbus, OH 43205, USA; The Ohio State University College of Medicine, Columbus, OH 43210, USA; Lawrence Berkeley National Laboratory, Berkeley, CA 94720, USA

## Abstract

Despite progress in the development of standards for describing and exchanging scientific information, the lack of easy-to-use standards for mapping between different representations of the same or similar objects in different databases poses a major impediment to data integration and interoperability. Mappings often lack the metadata needed to be correctly interpreted and applied. For example, are two terms equivalent or merely related? Are they narrow or broad matches? Or are they associated in some other way? Such relationships between the mapped terms are often not documented, which leads to incorrect assumptions and makes them hard to use in scenarios that require a high degree of precision (such as diagnostics or risk prediction). Furthermore, the lack of descriptions of how mappings were done makes it hard to combine and reconcile mappings, particularly curated and automated ones. We have developed the Simple Standard for Sharing Ontological Mappings (SSSOM) which addresses these problems by: (i) Introducing a machine-readable and extensible vocabulary to describe metadata that makes imprecision, inaccuracy and incompleteness in mappings explicit. (ii) Defining an easy-to-use simple table-based format that can be integrated into existing data science pipelines without the need to parse or query ontologies, and that integrates seamlessly with Linked Data principles. (iii) Implementing open and community-driven collaborative workflows that are designed to evolve the standard continuously to address changing requirements and mapping practices. (iv) Providing reference tools and software libraries for working with the standard. In this paper, we present the SSSOM standard, describe several use cases in detail and survey some of the existing work on standardizing the exchange of mappings, with the goal of making mappings Findable, Accessible, Interoperable and Reusable (FAIR). The SSSOM specification can be found at http://w3id.org/sssom/spec.

**Database URL**: http://w3id.org/sssom/spec

## Introduction

**Figure 1.**
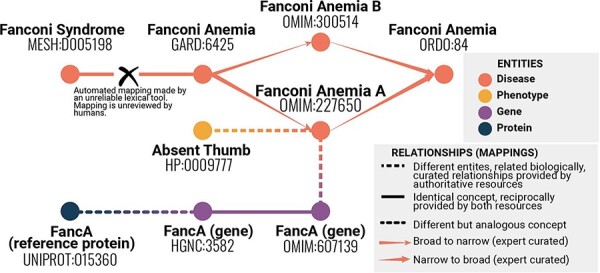
Example of mappings between different identifiers representing statements about similarity or identity of concepts across resources and vocabularies. Even with this simplified example, it is possible to see a range of mapping types, and that providing information about each mapping is crucial to understanding the bigger picture. This information helps avoid errors such as mistakenly conflating two variants of a disease.

The problem of mapping between different identifiers is ubiquitous in bioinformatics, and more generally in data science and data management. Equivalent concepts (entities) may be assigned different identifiers in different databases or vocabularies. Combining information from these multiple sources requires mappings between the identifiers. For example, a single gene or a single disease entity such as Fanconi anemia may be assigned different identifiers in different databases (Figure 1). If data from these databases are merged without mappings, then information related to the same entity, such as Fanconi anemia, is not combined, potentially losing crucial insights. Creating and maintaining mappings is costly, and the cost of incorrect or incomplete mappings can be even higher. For example, if health information is transferred between different systems, inaccurate mappings between disease terms could result in less accurate or even completely wrong diagnoses, with potentially serious negative consequences.

Despite their importance for data integration, term mappings are typically neglected as data artifacts (1). A mapping is a correspondence between two terms, referred to here as ‘subject’ and ‘object’ terms A ‘predicate’ defines the type of relationship between the subject and the object, such as skos:exactMatch, or owl:equivalentClass. A mapping, or ‘match’, does not have to be exact: it can be broad, e.g. between a conceptually narrow term such as ‘Red Delicious’ and a conceptually broader term such as ‘Apple’. Mappings can be defined between entities from different kinds of resources (e.g. from a database identifier to an ontology class), with ontological mapping relations ranging from vague cross-references to logical equivalence relations. Mappings are directional, i.e. they are defined in one direction (from ‘subject’ to ‘object’). Whether a mapping can be interpreted back (from the ‘object’ to the ‘subject’) is purely defined by the semantics of the predicate (e.g. owl:equivalentClass is symmetric as defined by the OWL specification). To our knowledge, no formal review has been published that analyzes the representation and formats used for collections of term mappings (mapping sets or alignments), but in our experience, most mapping sets are represented as tables using an ad hoc ‘schema’, often merely a simple two-column format that lists matching terms in two naming schemes, or alternatively as simple cross-references (without clear semantics) in ontologies themselves (2). The lack of metadata such as the semantics of the correspondence (is it exact?) or its provenance (was it reviewed by a domain expert?) makes it exceedingly difficult to reuse mappings and combine mappings from different resources. However, due to the often considerable cost involved in curating mappings, whether manually through domain experts or by automated tools, enabling mapping reuse is critical for many domains such as the biomedical and clinical.

Despite the importance of the mapping problem, there is no single widely agreed-upon standard for exchanging mappings. Existing schemes and formats frequently omit crucial information (see Table 1). For example, EDOAL (3), a widely used format in the Ontology Alignment Evaluation community, has never been adopted by the Open Biological and Biomedical (OBO) ontology community (4) because it lacked a sufficiently detailed metadata model. Many available mappings are just single-use conversion tables between two particular databases or database cross-references embedded in ontologies (5). These mappings generally have limitations: they are usually incomplete or inaccurate in ways that are nontransparent; they lack sufficient metadata to allow reuse in different contexts; and they do not follow FAIR (Findable, Accessible, Interoperable and Reusable) principles (6). Addressing these limitations is the central aim of the SSSOM standard.

**Table 1.** Desired features of a mapping standard, with examples of cases where the desired feature is met and examples where the desired feature is not met (negative examples)

Feature	Why	Examples	Negative example
Explicit relationship types	Applications that demand highly accurate results require mapping relations with explicit precision and semantics	EC:2.2.1.2 exactMatch GO:0004801 (transaldolase activity)	Two-column file that maps FMA ‘limb’ to Uberon ‘limb’, hiding differences in species-specificity
Explicit confidence	Different use cases require different levels of confidence and accuracy	A mapping tool assigns a confidence score based on the amount of evidence that is explicitly recorded	Without the confidence score we cannot filter out automated mappings with low confidence
Provenance	Understanding how a mapping was created (e.g. automatically or by a human expert curator) is crucial to interpreting it	Mapping file that automated mappings with link to tool used; curated mapping file with curators’ ORCIDs provided	Two-column mapping file with no indication of how the mapping was made, and no supplementary metadata file
Explicit declaration of completeness	Must be able to distinguish between absence due to lack of information vs deliberate omission	Mapping file where rejected mappings are explicitly recorded	Mapping file where absence of a mapping can mean either explicitly rejected mapping OR the mapping was not considered/ reviewed
FAIR principles	Mappings should be Findable, Accessible, Interoperable and Reusable	Mapping file available on the web with clear licensing conditions, in standard format, with full metadata and a persistent identifier	Mapping files exchanged via email
Unambiguous identifiers	Mapping should make use of standard, globally unambiguous identifiers such as CURIEs or IRIs	Standard ontology CURIEs like UBERON:0002101 for entities, with prefixes registered in a registry or as part of the metadata	Identifiers are used without explicitly defined prefixes; mappings are created between strings rather than identifiers
Allows composability	Mappings from different sources should be combinable and should be possible to chain mappings together	Defined mapping predicates (relations) such that reasoning about chains A-> B-> C is possible (where allowed by semantics of the predicate)	Two mapping files with implicit or undefined relationships -> unclear whether these can be combined or composed
Follows Linked Data principles	Allows interoperation with semantic data tooling, facilitates data merging	All mapped entities have URIs, and metadata elements also have defined URIs; available in JSON-LD/RDF	No reuse of existing vocabularies for metadata or for relating mapped entities
Well-described data model	Allows interoperation and standard tooling	Data model provided in both human and machine-readable form	Ad hoc file format with unclear semantics
Tabular representation	Ease of curation and rapid analysis	A mapping available as a TSV that is directly usable in common data science frameworks; may complement a richer serialization	Ad hoc flat-file format requiring a custom parser

### Desired features of a standard for mappings

We cataloged key characteristics of standard mappings, based on the diverse group of use cases described later in this paper. We usually refer to an entity that describes the relationship as the ‘predicate’, but generally use the terms ‘predicate’ and ‘relationship’ loosely to mean the same thing.

These features include explicitly declaring the relationship between the two mapped entities. Frequently mappings are released as simple two-column files with no information about how the entities are related. Many applications benefit from or require mappings to be categorized as to whether the mappings are exact, or whether one concept is more general than the other, versus being closely related, but neither exact nor broader/narrower. There are a variety of different vocabularies that can be used to describe the relationship, including the Simple Knowledge Organization System (SKOS) (7) and the Web Ontology Language (OWL) (8), with different use cases dictating which system is used.

Additional desirable characteristics include various pieces of metadata associated with either a mapping collection or individual mappings, describing the provenance of the mapping (who made it, what tool made it if automated, when it was made), versioning, indications of confidence and completeness. This information helps humans understand and interpret the mappings, and can also be used by software.

We also include in our list of desiderata adherence to FAIR principles (6) and Linked Data principles (9). Linked Data principles aim to make data interoperable through the use of common data formats such as RDF and Uniform Resource Identifiers (URIs) for naming and identifying individual things. This includes making mappings easily available on the web as well as using standard URIs for representing both mapped entities and mapping data elements. There should also be a well-defined data model. Additionally, there should be a simple tabular form to enable easy exploration, management, viewing and processing by computational and human users, without needing specialized editing tools.

*Our solution: In this paper, we present SSSOM, a Simple Standard for Sharing Ontological Mappings (pronounced ‘sessom’ or S.S.S.O.M). SSSOM’s goals are:*

1. Providing a rich and easily extensible vocabulary for describing mapping metadata to address the aforementioned issues by encouraging the publication of mappings that are transparently imprecise, transparently inaccurate and transparently incomplete, as well as FAIR.
2. Offering a simple tabular format for the dissemination of mappings that can be easily integrated in typical data science toolchains.
3. Supporting a community-driven standard with well-defined governance and sustainable collaborative workflows.
4. Representing many different kinds of mappings, such as mappings between data models and their values, including literal values, controlled vocabularies and database entities.

### SSSOM: a rich and extensible vocabulary and schema for mapping metadata

In this section we describe the SSSOM standard in four subsections:

- The core data model and the metadata elements included in SSSOM
- How SSSOM is exchanged, including the canonical simple tabular serialization
- Governance and sustainability of the standard
- The emerging software ecosystem for working with SSSOM mappings

The complete SSSOM documentation and specification can always be retrieved via a permanent URL using the w3id system, https://w3id.org/sssom/ (10), and project information and source schema files can be found in our GitHub repository (https://github.com/mapping-commons/sssom). The current version of SSSOM at the time of this writing is 0.9 (11). A detailed description can be found in the online documentation (10), but we will discuss many of the key features and their rationale later in this section.

At heart, SSSOM is a simple data model for representing mappings and mapping set metadata. ‘Simple’ in this context means ‘flat’, i.e. suitable for describing data that is primarily exchanged in a tabular form such as TSV or CSV, as opposed to JSON, which allows for nested data structures. This simplicity, although it presents limitations (see section on Limitations), is one of the central design principles: the more complex structures like nested metadata or expressions (e.g. ‘limb part’ in one resource maps to the OWL expression ‘“part of” some “limb”’ in another) we allow, the more error-prone published mapping sets will become, and the more dependent we make users of the SSSOM standard on specific toolkits and software libraries—something we want to avoid as much as possible. Equally important, despite emerging toolkits for curating mappings, it is our experience that most mapping sets (certainly the ones used across all projects the authors are associated with) are curated as tables, which we will discuss later in this section. Despite this strong emphasis on simplicity, we are currently drafting a proposal to allow more deeply nested metadata (for example multiple mapping fields) and complex expressions (see Discussion section) through ‘profiles’ that can be built on top of the current simple standard.

### Data model

The SSSOM data model describes individual pairwise mappings, which are grouped into mapping sets.

Each mapping can be described by up to 38 standard metadata ‘slots’, or elements (in version 0.9). Four of these are required for any individual mapping: subject_id, object_id (the pair of entities mapped), predicate_id (the nature of the relationship between the two) and match_type (how the mapping was derived). Additional optional metadata elements include author_id, mapping_date and many more. For mapping sets, there are 23 elements, including mapping_set_id, license and creator_id.

All identifiers used in SSSOM should be CURIEs (12), i.e. prefixed identifiers with a registered prefix, following identifier best practice (13). Thirteen SSSOM elements are currently mapped to external vocabularies. For example, author_id and mapping_date are mapped to PAV (14) publication_date, license and others are mapped to Dublin Core (15). These mapped predicates are used in the RDF and JSONLD serializations of SSSOM.

**Figure 2.**
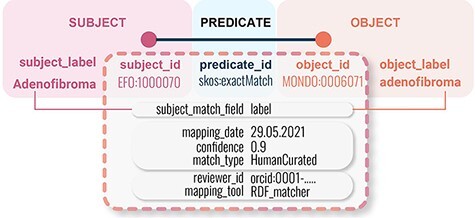
Example of basic SSSOM mapping model with some illustrative mapping metadata elements.

An example mapping with a few select metadata elements can be seen in Figure 2.

### Predicates

SSSOM allows any vocabulary to be used to describe the relationship (predicate) between subject and object, but we recommend that the predicate_id is drawn from either SKOS or OWL vocabularies, in particular one of the predicates listed in Table 2.

**Table 2.** Recommended values of predicate_id capturing a broad range of use cases, drawn from SKOS vocabularies and from OWL

Predicate	Description
owl:sameAs	The subject and the object are instances (OWL individuals), and the two instances are the same.
owl:equivalentClass	The subject and the object are classes (OWL class), and the two classes are the same.
owl:equivalentProperty	The subject and the object are properties (OWL object, data, annotation properties), and the two properties are the same.
rdfs:subClassOf	The subject and the object are classes (OWL class), and the subject is a subclass of the object.
rdfs:subPropertyOf	The subject and the object are properties (OWL object, data, annotation properties), and the subject is a subproperty of the object.
skos:relatedMatch	The subject and the object are associated in some unspecified way.
skos:closeMatch	The subject and the object are sufficiently similar that they can be used interchangeably in some information retrieval applications.
skos:exactMatch	The subject and the object can, with a high degree of confidence, be used interchangeably across a wide range of information retrieval applications.
skos:narrowMatch	The object of the triple is a narrower concept than the subject of the triple.
skos:broadMatch	The object of the triple is a broader concept than the subject of the triple.

‘match_type’ is a term from a controlled vocabulary that describes the method by which the match was established that led to the mapping. There are currently five types of matches in SSSOM:

1. Lexical: the match was determined through a lexical analysis of some kind.
2. Logical: the match was determined by an automated reasoner (16).
3. HumanCurated: the match was determined by a human expert.
4. SemanticSimilarity: the match was determined by a semantic similarity algorithm such as Resnik or Jaccard (17).
5. Complex: the match was determined by a variety of strategies, usually as part of an automated matching tool.

Each of these match types can be refined through a combination of other metadata elements. For example, a lexical match should be further qualified using the subject and object ‘match_field’. The match field can be set to the CURIE for the property that was used to perform the match. This can be a property from a standard vocabulary such as SKOS, Dublin Core (OMO) (18) or RDFS, for example:

- rdfs:label, when the match is on the primary label for the matched entity
- skos:exactMatch, when the match is on a common matched entity
- oboInOwl:hasExactSynonym, when the match is to an exact synonym of the entity

Lastly, if the match occurred after applying preprocessing, for example stemming or lemmatization, this can be captured by the ‘preprocessing’ metadata field. Semantic similarity matches can be further refined by providing a ‘semantic_similarity_score’ and ‘semantic_similarity_measure’. All automated matches, in particular complex matches, should make reference to a ‘mapping_tool’ and its ‘mapping_tool_version’.

### Provenance

Most SSSOM metadata elements pertain to provenance. We will describe some of the most important ones here, and refer the interested reader to the full list in the online documentation (19). Mappings are maintained and established by authors (‘author_id’), owned and published (i.e. brought into their SSSOM mapping form) by creators (‘creator_id’), and reviewed by one or more reviewers (‘reviewer_id’). For maximum transparency we recommend the use of ORCID CURIEs (20), ROR IDs (21) for organizations and Wikidata IDs (22). For example, a domain expert (orcid:0000‐0002‐7356‐1779) determines that UBERON:0002101 (limb), is an exact match (i.e. skos:exactMatch) to the term FMA:24 875 (‘Free limb’). Domain expert orcid:0000‐0002‐7356‐1779 is a consultant for the European Bioinformatics Institute (EMBL-EBI, ror:02catss52), which produces the SSSOM mapping set that the above mapping is captured in and publishes it. Curator orcid:0000‐0002‐7073‐9172 reviews the mapping and confirms it.

The subject and object of a mapping each come from a source, such as an ontology or a database (‘subject_source’, ‘object_source’). For example, the term UBERON:0002101 comes from a source ‘Uberon’. When the mapping is created, it is usually based on a specific version of the source (e.g. ‘subject_source_version’) which we recommend encoding with a version string such as ‘1 January 2020’ or ‘2.1.0’. This is important, especially for making incompleteness transparent—potentially missing mappings can now be attributed to an outdated mapping set. The mapping set itself is similarly attributed an ID (‘mapping_set_id’) and version (‘mapping_set_version’).

Finally, the ‘mapping_date’ is the date on which the mapping was established by the mapping author, and the ‘publication_date’ is the date on which the SSSOM mapping file was published by its creator. The ‘why’ and ‘what’ of provenance are implicit in the model. For the ‘why’, we expect that the intention is to map two entities in an unconditional fashion, i.e. that we model the case where the mapping is always true; see ‘Limitations’ section. Any contextual parameters that need to be considered when interpreting the mapping should be explicit in the mapping predicate.

### LinkML specification

The SSSOM schema is managed as a LinkML (23) model. LinkML, the Linked Data Modeling Language, allows schemas describing the structure of the data to be authored in YAML format. LinkML gives us a range of advantages for managing our schema:

1. We can automatically convert it into common schema representations such as JSON Schema, ShEx, SHACL or OWL (these are all available from the GitHub repository).
2. We can use LinkML utility classes to automatically convert instance data into common representations such as JSON or RDF.
3. We can use LinkML meta models to automatically generate Python dataclasses and implement data validators etc., and we use these in our own Python toolkits (see below).
4. The SSSOM schema in YAML is easier for domain experts to read and maintain, compared to other complex schema representation languages such as OWL or JSON Schema.

### SSSOM TSV format: a simple tabular format for dissemination of mappings

A simple, table-based serialization of mapping sets was one of the core requirements for creating SSSOM. Tables are, in our experience, by far the most widely used data source in data science pipelines, and still the preferred medium for curating data. Many related approaches in the Semantic Web community such as those discussed as part of the ‘Semantic Web Challenge on Tabular Data to Knowledge Graph Matching’ [SemTab (24)], reflect the importance of tables as a mechanism for curating data. Therefore, SSSOM TSV format should be considered the native SSSOM data format, with other formats like RDF/XML or JSON-LD functioning as export formats. The reason a ‘native’ format is important is that we do not only want to offer a better model for capturing metadata but also promote better practices for mapping as a process. One of these practices is that we want to produce mappings that are consumable and interpretable by very general toolchains, such as the ones used in data science [Pandas (25)], etc. While we do provide a Python toolkit for the more advanced operations involving SSSOM mapping sets, it was a key design consideration that SSSOM mapping files should be readable by general toolkits, without the need of any special tooling.

**Figure 3.**
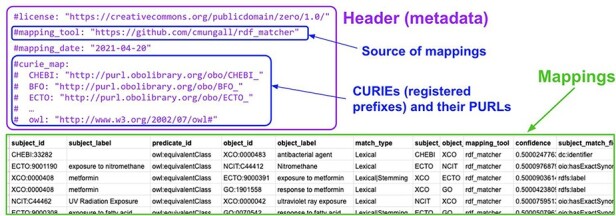
An example SSSOM TSV table (generated by the developers of the environmental exposure ontology (19) using rdf-matcher (26)), with a table header (lines that start with #, shown in purple) that contains the mapping set metadata, followed by the mappings (27).

A SSSOM TSV table comprises two main parts: the actual table which contains the mapping and its metadata, and a table header which contains the mapping set metadata. Figure 3 shows an example of part of a simple SSSOM TSV file. The header part of the table is commented YAML (indicated by the leading # symbol). For practical purposes, we support both this ‘embedded’ mode, where the YAML header is provided together with the mapping table and an ‘external’ mode, where the SSSOM YAML header is supplied as a separate file. Due to the risks involved in managing two files (losing provenance during sharing, etc.), we promote the use of the embedded mode, but the SSSOM Python toolkit can convert from external to embedded mode to ensure compatibility.

### Sustainability: collaborative workflows and governance

The SSSOM standard is maintained as an open-source project on GitHub in the mapping-commons organization. No single organization is responsible for the sustainability of the SSSOM standard, but a number of organizations (see Funding) are providing core funding for its development. Once the SSSOM standard is fully defined, few resources aside from web hosting will be required to sustain it. During our inaugural workshop in September 2021 (28), we established our basic governance rules (https://github.com/mapping-commons/sssom/issues/82). We make heavy use of GitHub collaborative workflows including issue templates, pull requests and reviews, GitHub actions for Continuous Integration and, perhaps most importantly, a public issue tracker to respond to and manage our interactions with the wider mapping community.

Changing the schema. The SSSOM schema is managed entirely as a LinkML model (23), with the source YAML file managed in GitHub. To change the schema, we perform the following actions. For every change (usually adding/changing metadata elements), we require the creation of a GitHub issue detailing the nature of the change. This ensures that the community has time to respond to the intended change even before it is performed. If the community reaches an agreement on the nature of the change, an edit to the source schema is created and a GitHub pull request is opened. The pull request stays open for review. If the schema change is not backward compatible (i.e. current SSSOM mappings are affected), the change needs to be approved by members of the core team.

### The SSSOM software ecosystem

There are several useful tools for working with SSSOM. sssom-py is a Python library and a command-line toolkit that was designed to work with SSSOM (29). The library covers functionality such as importing files from different formats [OBO Graphs JSON (30), RDF Alignment API (31)] and exporting them as SSSOM tables; converting SSSOM tables to RDF, OWL (a variant of RDF that includes entity declarations required for conformance with the OWL standard) or JSON-LD; merging and querying SSSOM tables and validating them. For an overview of the full functionality of sssom-py, refer to the documentation (32). Extracting SSSOM tables from ontologies should make it easier for ontology developers that prefer to curate their mappings as part of their ontology. rdf-matcher is a matcher for RDF vocabularies or OWL ontologies that exports mapping sets as SSSOM tables, including mapping rules (26). For example, rdf-matcher exports metadata such as mapping tool, confidence, match fields and match string. It can document simple mapping rules such as matches on label and synonym fields.

In our vision for the publication of terminological mappings, related mapping sets are collected and even maintained as part of a mapping commons. A mapping commons is a public registry that enables users to find mappings for a clearly defined use case such as ‘cross-species phenotype mappings’ or ‘disease mappings’. An example of a mapping commons (which is entirely independent of the SSSOM standard and its core team) that focuses on mappings related to mice and humans can be found on GitHub (33). The creation of mapping commons is in the very early stages, but the hope is that users can simply report wrong mappings much the same way as they can document issues on other semantic artifacts such as ontologies or terminologies.

### Why we need better metadata for terminological mappings: use cases

#### Here we describe four use cases that motivated the development of SSSOM

##### Use Case 1: harmonizing disease mappings: Mondo Disease Ontology

The Mondo disease ontology (34) seeks to harmonize a variety of disease ontologies and terminologies in a consistent logical framework. Mondo not only provides semantically precise mappings to external sources; it also ensures that these mappings are reconciled, i.e. no single external term will ever map to more than one term in Mondo. This enables users to map their disease data to Mondo from a variety of sources such as Online Mendelian Inheritance in Man [OMIM (35)], Disease Ontology [DO (36)], Orphanet (37), National Cancer Institute Taxonomy [NCIT (38)] and the International Classification of Diseases [ICD (39)], and analyze the data in a coherent logical framework.

Maintaining a harmonized set of mappings is a complex task. To make the integration of more terminological sources and the ongoing maintenance of mappings scalable, Mondo uses an automated Bayesian approach for ontology merging [k-BOOM (40)], which takes as an input the two ontologies to be aligned and a set of mappings with probabilities. These mapping sets can be generated by any matching tool, as long as there is some kind of confidence/probability score and a precise mapping predicate (e.g. skos:exactMatch, skos:narrowMatch, etc.). The current implementation of the k-BOOM algorithm in the Boomer tool (41) reads SSSOM files as mapping candidates and then discovers the most likely ‘correct’ mappings. Tools like Boomer rely on mappings with transparent imprecision and accuracy to work effectively.

In addition to maintaining a set of harmonized mappings, Mondo also has to distribute them. Before SSSOM, mappings were primarily distributed as owl:equivalentClass axioms and skos:exactMatch (or even oboInOwl:hasDbXref) annotations, which made them hard to use for any but ardent users of semantic web technologies. Mondo now exports SSSOM tables as part of their release pipeline. Because these tables include explicit provenance information, they allow downstream users to use the mappings effectively.

##### Use Case 2: browsing and cross-walking mappings: O×O

The European Bioinformatics Institute (EBI) developed the Ontology Xref (Cross-reference) Service [O×O (42)], to enable users to find suitable mappings for their ontology terms and provide APIs to access them (43). O×O integrates cross-references from OBO ontologies and mappings from UMLS and other sources. Users make heavy use of O×O’s ability to ‘walk’ mappings. ‘Walking’ (also known as cross-walking or hopping) is the ability to link terms together based on intermediate mappings. For example, a user might look for suitable mappings for FMA:24 875 (‘Free limb’), e.g. https://www.ebi.ac.uk/spot/oxo/terms/FMA:24875. Within mapping distance 1 (1 hop) we only find a single suitable match at the time of this writing (October 2021), UBERON:0002101 (‘limb’). If we increase the search radius to mapping distance 2, we find seven additional mappings which look fine, like MA:0000007 (limb) or NCIT:C12429 (Limb). However, we also see the first issues emerge: EFO:0000876 (obsolete vertebrate limb) and UMLS:C0015385 (Extremities) are also among the search results. Terms that are marked as obsolete should not be used in mappings, and the term ‘extremities’ usually refers to appendages such as hands or feet rather than the whole limb. Indeed, on closer inspection, we find that ‘Extremities’ is mapped to UBERON:0000026 (appendage) in O×O. A blind application of walks cannot work if we do not know that the mapping from ‘limb’ to ‘extremities’ is related rather than exact—rather than being simply ‘cross-references’ without precise semantics, we need our mappings to be transparent about imprecision. O×O was designed as a tool to query and walk ‘cross-references’, encoded in ontologies as hasDbXrefs, which do not have ‘precision’ by design—they often correspond to exact matches, but they can correspond to broad, narrow, close or related matches, without explicitly specifying that as part of the metadata. This captures the original use case of O×O: finding closely related terms across terminologies and ontologies. With the advent of SSSOM, O×O seeks to enable further use cases, like cross-walks with precise mappings, by capturing additional metadata. A first draft of this extension to the current O×O data model and a prototype user interface is planned for May 2022.

Users want to be able to view only the trustworthy mappings, and what we deem ‘trustworthy’ is very much dependent on our personal experience and preference. While it is already possible to restrict search in O×O to particular sources, O×O imports all cross-references found in these sources, disregarding any additional metadata. For example, unlike Mondo (described in Use Case 1), O×O currently does not distinguish between skos:exactMatch and skos:relatedMatch. To convince users that a particular mapping is good enough for their particular use case, we may need to present the mapping rules that were applied to determine the mappings. Such metadata does not currently exist at all in most mapping sets, but in order to curate and then leverage it, we must first provide standards like SSSOM to represent common mapping rules, which can then be implemented by tools like O×O.

##### Use Case 3: National Microbiome Data Collaborative

The National Microbiome Data Collaborative [NMDC (44)] integrates environmental omics-related data and metadata from multiple sources. This involves aligning metadata schemas from multiple different sources including the Genomes OnLine Database (GOLD), NCBI, the NMDC schema and the Genomics Standards Consortium Minimum Information about any (x) Sequence [MIxS (45)] standard. It also involves aligning underlying vocabularies used to describe categorical aspects of samples, including the GOLD environmental path vocabulary and the Environment Ontology (ENVO) (46).

NMDC has created SSSOM files for these mappings, making use of multiple aspects of the SSSOM standard, including the mapping predicate (most mappings are exact, but a small handful are related matches), and whether the mapping has been curated by an expert or was obtained from a specific source. Using SSSOM allows the NMDC to use standard tools for summarizing and validating these mappings.

##### Use Case 4: finding and using mappings in EOSC-Life

The EOSC-Life project (​​https://www.eosc-life.eu/) (47) brings together the 13 Biological and Medical ESFRI research infrastructures to create an open collaborative space for digital biology in Europe. EOSC-Life has designed a use case with ‘Alice’ (a fictional use case persona) as a data steward who needs to register patient information in the European registry for Osteogenesis imperfecta, which uses Orphacodes (from Orphanet) for diseases, whereas other partners use SNOMED CT. To demonstrate the automatic conversion from SNOMED CT to Orphacodes, they set up a FAIR Data Point (FDP) with the metadata description of the mappings and used SSSOM to describe mappings. FDP is a realization of FAIR data principles that stores database metadata and publishes it on the web. Compared with the simple equivalence between the objects of the same subject provided by other mapping systems, mappings described by SSSOM have richer metadata, e.g. specifying match precision. Combining FDP and SSSOM, it is possible for Alice to access mappings via a FDP according to their semantics, and to automatically use the mappings by converting specified subjects to the mapped objects accurately via SSSOM metadata.

### Related work

In this section, we discuss alternative formats for capturing terminological mappings, as well as some less formal efforts concerned with mapping metadata. We also describe some of the impactful mapping-related tools and discuss how they could benefit from implementing a standard mapping metadata model such as SSSOM.

### Standard formats for capturing mappings

The Ontology Alignment Evaluation Initiative [OAEI (48–50)] is a coordinated international initiative to forge consensus for evaluation of ontology matching methods. OAEI participants such as Agreement Maker Light (51) and LogMap (52) have played a significant role in improving automated mappings. The RDF Alignment Format (3) is currently the main format used to exchange mappings within the ontology matching community and the OAEI. The main advantage of the RDF Alignment Format is its simplicity. EDOAL (3) is a more expressive format that aims at representing complex mapping, e.g. linking two or more entities beyond atomic subsumption and equivalence. Both the RDF Alignment and EDOAL formats are supported by the Alignment API (53). SSSOM, like the RDF Alignment Format, brings a simple format to exchange mappings, which improves on the metadata and provenance information associated with the mappings, enhancing their understanding and potential future reuse. The Matching Evaluation Toolkit [MELT (54)] is a framework for developing, tuning, evaluating and packaging ontology matching systems that has been adopted by OAEI. Currently MELT supports the RDF Alignment and EDOAL formats, but it is a modular framework that can easily support additional mapping exchange formats like SSSOM. The organizers of the yearly OAEI evaluation event are considering adopting SSSOM as an additional mapping exchange format.

The Vocabulary of Interlinked Datasets [VoID (55)] is a W3C Interest Group RDF Schema vocabulary for expressing metadata about RDF datasets (56). Beyond describing RDF datasets in general, VoID allows the specification of Linksets, i.e. collections of links where the subject is in a different dataset than the object. VoID metadata elements are fairly high level and need to be extended to capture fine-grained provenance and mapping rules. The Open PHACTS project extended VoID, in particular, to support mapping justifications using the BridgeDb Mapping Vocabulary (57). BridgeDb (58) is an open-source data identifier mapping service that is typically used for mappings between genes and gene products, metabolites and reactions. In principle BridgeDb can also provide ontology mappings; for instance, it has already been used for gene–disease relationships. BridgeDb can stack mappings; the most common use case for that is when people use their own ontology or identifier class and want to map these first to an external identifier and then to relate them using standard mappings. A semantic web version of BridgeDb was developed as the OpenPHACTS Identifier Mapping Service (59). We are discussing integrating VoID linksets and the BridgeDb mapping vocabulary with SSSOM mappings mapping sets, which provide richer metadata. However, some obstacles exist. For example, the VoID specification does not permit multiple subjects or objects in a single linkset file, which is a critical requirement of SSSOM.

Some mapping metadata can be captured using simple established vocabularies such as Dublin Core (15) and OWL (8). There are also a variety of approaches to capturing more detailed provenance, such as PROV-O (https://www.w3.org/TR/prov-o/), PAV (https://pav-ontology.github.io/pav/) and the Mapping Quality Vocabulary (MQV) (https://alex-randles.github.io/MQV/). The SSSOM data model allows some basic provenance information to be captured using properties such as creator_id and mapping_provider. Currently, two SSSOM properties are mapped directly to PAV and one to PROV-O but work is underway to provide a complete mapping to these established standard vocabularies, including a more comprehensive alignment with the PROV-O activity model.

The Distributed Ontology, Modeling and Specification Language [DOL (60)] is an Object Management Group [OMG (61)] standard for the representation of distributed knowledge, system specification and model-driven development across multiple ontologies, specifications and models (OMS). DOL enables the representation of alignments across OMS that have been formalized in different formal (logical) languages on a sound and formal semantic basis. In contrast to SSSOM, DOL deals primarily with distributed semantics and does not define a vocabulary for mapping metadata and mapping rules. Another OMG standard that includes a component for terminological mappings is the Common Terminology Services 2 System [CTS2 (62)]. CTS2 supports the management, maintenance and interaction with ontologies and medical vocabulary systems, providing a standard service information and computational model. The CTS2 Map Services specify how entity references from one code system or value set are mapped to another. The CTS2 map entry information model reflects many of the metadata elements also defined by SSSOM, such as subject and object source references, version information and mapping set names. In contrast to SSSOM, CTS2 allows mapping one entity to multiple targets in complex mapping expressions and specifies the expected behavior of mapping services. Overall, it is considerably more complex than SSSOM and geared toward interoperability between software systems in a wider clinical context rather than FAIR exchange of terminological mapping.

​The Systematized Nomenclature of Medicine—Clinical Terms (SNOMED CT) is a clinical terminology designed to represent content in electronic health records (63). The SNOMED CT logical model, unlike SSSOM, does not require extensive provenance on how a mapping was created. Another difference between these resources is transparency and accessibility. Currently, the mappings provided by SNOMED CT must be built locally using their OTF-Mapping-Service (https://github.com/IHTSDO/OTF-Mapping-Service). SSSOM is a fundamental component underlying all of the Mapping Commons (https://github.com/mapping-commons), which means all projects within the Commons are interoperable and publicly available. Perhaps the most important distinction is that unlike the SNOMED CT Reference Sets, which are explicitly designed for use with SNOMED-specific resources, SSSOM is not designed for use with a single system, infrastructure or standard.

The Unified Medical Language System (UMLS) (64) is a set of resources including a repository of biomedical vocabularies and terminologies developed by the US National Library of Medicine (NLM). The NLM coordinates a number of mapping efforts, such as SNOMED to ICD 10 (65). UMLS maps 214 vocabularies based on automated approaches that exploit lexical and semantic processing and manual curation (66). The UMLS API can exploit mappings to enable cross-walks, much the same way as O×O does (see Section on Use Cases). While the UMLS mapping model [MRMAP (67)] is probably the closest to a standard tabular representation for mapping metadata, it lacks many of the metadata elements defined by SSSOM, and, more importantly, does not define a public, collaborative workflow for defining new metadata elements or formally defining mappings into other formats such as RDF or JSON.

### Informal approaches for capturing mappings

In addition to the mapping standards described above, there have been various less formal attempts to capture mappings. Some of these were launched to address a specific need but fail to address some of the requirements that SSSOM satisfies. Many ontologies in OBO (4) make use of the oboInOwl:hasDbXref property (68), also known as the ‘database cross reference’, as historically most mappings have been created as simple cross-references in this fashion. A drawback of these ‘xref’ mappings is that they are semantically ambiguous and are used in vastly different ways in different ontologies (2). After analyzing about a million such database cross-references across OBO ontologies, Laadhar *et al.* concluded that their unclear semantics makes them ‘impractical or even impossible to reuse’—a viewpoint that the authors of this paper share.

For the BioHackathon 2015, members of the DisGeNET team (69) and their collaborators surveyed a number of sources to define a minimal set of attributes and standards for ontology mapping metadata (70). The BioHackathon 2015 never resulted in a formal specification for mapping metadata, but we are now working with the DisGeNET team to incorporate the metadata elements of their survey directly into SSSOM. Most of their proposed metadata elements have already been mapped to SSSOM; others are being currently revised.

The Semantic Mapping Framework (SEMAF) is a European-Commission-funded study designed to formalize a framework to create, document and publish FAIR mappings between semantic artifacts, e.g. vocabularies, ontologies and lexicons, used in multiple scientific domains (1). To understand the limitations of existing mapping approaches and develop reasonable solutions, the SEMAF Task Force conducted interviews with experts in a variety of communities, including 25 experts from a wide range of scientific domains, and reviewed 75 reports on existing research infrastructure (71). From this work, the SEMAF Task Force identified an extensive set of requirements that span infrastructure, architecture, data models, user interfaces, machine access, optional and content management and implementation. Their framework, which consists of a Federative Registry and a Mapping Model, was designed to support these requirements. There are many aspects of the SEMAF Mapping Model that align with the Mapping Commons principles and with SSSOM. Both SSSOM and SEMAF are based on Semantic Web principles and use assertions to provide additional metadata about a mapping, e.g. mapping provider, creation date. As the SEMAF model is still emerging, it is not yet clear how exactly it will be implemented (schemas, toolkits) and which metadata elements will be included. It is, however, our understanding from the current documentation that SSSOM would be a suitable implementation for the abstract SEMAF mapping model.

OMOP2OBO (https://github.com/callahantiff/OMOP2OBO) is the first health system-wide semantic integration and alignment between the Observational Health Data Sciences and Informatics’ Observational Medical Outcomes Partnership (OMOP) standardized clinical terminologies and OBO biomedical ontologies (72). The OMOP2OBO framework provides both a mapping algorithm and an open-source repository of mappings. OMOP2OBO uses a sophisticated mechanism for converting flat-file mappings into RDF and OWL (73), which is currently being aligned with SSSOM.

### Mapping services and tools

The EMBL-EBI Ontology Xref Service (O×O) (https://www.ebi.ac.uk/spot/oxo/) provides a web-based user interface and REST API to allow retrieval of mappings between terms We are working with the O×O team to extend their mapping model to support SSSOM natively, (see Section on Use Cases). This will make the output of O×O more useful by including further information about mappings and allow O×O to ingest mappings from any SSSOM datasource.

The BioPortal software (along with its deployed versions based on the OntoPortal distribution) manages mappings of multiple types from a variety of sources (74, 75) and presents them in two contexts (ontology-to-ontology and term-to-term) and via two access methods (UI and API). Types of mappings include URI (same IRI in both places), CUI (matching Concept Unique Identifiers (CUI) values in UMLS terms), LOOM [a syntactical match using the LOOM algorithm (76)] and REST (mappings provided by BioPortal users). BioPortal automatically creates the URI, CUI and LOOM mapping information each time an ontology is updated. Several metadata attributes are stored with each mapping, including a timestamp, the process that created the mapping (including the user who provided each REST mapping), the mapping relationship and mapping type. As with O×O, a user submitting REST mappings to BioPortal must convert their data to the defined BioPortal mapping submission format. The BioPortal team intends to provide SSSOM support and a SPARQL endpoint and provide this code as part of its shared OntoPortal Appliance distribution, used by repositories such as AgroPortal and EcoPortal.

Biomappings (77) is a repository for both curated and predicted mappings along with their associated metadata. It is intended to fill in the gaps in the availability of mappings between widely used resources. Biomappings is built on public tools such as git and GitHub, uses automated testing and continuous integration to check data integrity, provides a web-based curation interface for triaging predicted mappings and adding novel ones and offers several workflow examples for generating new predictions using Gilda (78) or custom scripts. Its data are available under the CC0 1.0 license and distributed in the SSSOM format to promote contributions, reuse and enable incorporation into primary resources.

The Pistoia Alliance best practice guidelines were designed to check how suitable source ontologies are for mapping (79). They emphasize the application of ontologies in the life sciences to encourage best practices and aid mapping of ontologies in a particular domain. This public resource was developed as part of the Pistoia Alliance Ontologies Mapping project, which also defined the requirements for an Ontologies Mapping tool and service (80, 81). This led to the development of Paxo (82), a lightweight Ontology Mapping tool designed to align ontologies hosted by the OLS (83) and to integrate them with O×O (43). The alignments generated from Paxo and O×O are available, but they are currently limited to CSV format. These mapping alignments would benefit greatly from transformation into the much more expressive SSSOM format to capture the relevant metadata, and some of them have already been converted (https://github.com/mapping-commons/mh_mapping_initiative).

## Discussion and limitations of the approach

In this section, we discuss shortcomings of the current SSSOM approach and potential ways to address them:

1. Mappings themselves have no context (i.e. are always true)
2. Complex mapping rules are hard to capture due to the simple, flat data model
3. Mappings are not idempotent, i.e. there are metadata elements that modify one another
4. Lack of support for complex mappings

Mappings have no (global) context. There are many mapping scenarios, especially in the clinical domain, where mappings only hold under a range of applicability criteria. For example, we could say that ‘UBERON:0002101 (metazoan limb) is equivalent to XAO:0003027 (xenopus limb) under the assumption that taxon constraints are ignored. Or we might want to express that you can swap one term from a clinical terminology for another, but only if we can assume the patient is an adult female. It was an important design decision for SSSOM to decide that mappings should be universally applicable and not dependent on some global context, which would make merging and reconciling them much more complex (requiring specialized tooling). While this can be a significant problem for some use cases, there are two potential workarounds (one that is currently supported and one that is currently under discussion) (1): the contextual parameters of the mapping can be captured as part of the mapping relation. For example, one could define a new relation ‘example:hasExactCrossSpeciesMatch’ as a sub-relation of skos:closeMatch that links UBERON:0002101 and XAO:0003027. The problem with this approach is, while currently supported, it would push the contextual parameters far away into an ontology of relations, which mapping applications, for example, would have to import and interpret (2). The contextual parameters could be captured as complex expressions. For example, you could define UBERON:0002101 (limb) + NCBITaxon:8353 (xenopus) → XAO:0003027 (xenopus limb), and capture ‘UBERON:0002101 (limb) + NCBITaxon:8353 (xenopus)’ as an ontological class expression such as ‘UBERON:0002101 and “in-taxon” some NCBITaxon:8353’. This is currently not supported, but is being discussed. Ultimately the balance here is between capturing all mapping scenarios and keeping the metadata and format as simple as possible. It seems to be the case that a large percentage of use cases can be captured without introducing complex expressions.

Complex mapping rules are hard to capture in a simple, flat model. Many matching decisions, in particular those done by automated tools, are complex: they involve a variety of mapping rules. For example, an automated matching tool may determine that based on a specific threshold of semantic similarity, e.g. > 0.9, and a matching label, we decide that the subject-predicate-object triple constitutes a match. A flat data model like SSSOM cannot easily capture the case where a match is associated with multiple match types. Again, this modeling decision comes down to the simplicity vs. expressivity tradeoff described above. While it would be easy to build a data model that supports multiple complex match types, it violates one of our central requirements: being able to express the data as a simple table. We, therefore, decided to accept this shortcoming. For our use cases that require complex match types, we, therefore, provide ‘one row per mapping rule’. Our reference implementation, rdf-matcher, for example, would produce two rows for the match between UBERON:0002101 and FMA:24 875 if there was a lexical match on an exact synonym of the terms and also a lexical match on the primary labels of the terms.

Mappings are not idempotent: adding a column to a mapping table could change its semantics. The hardest design decision to make was regarding the modifiers on ‘predicate_id’. There are many use cases for modifiers, such as negation: you want to be able to say that UBERON:0002101 is NOT a skos:exactMatch to FMA:54 448. After debates during our first Workshop on SSSOM (28), we decided to add a ‘predicate_modifier’ element to SSSOM which allows such encodings. The alternative would have been to introduce additional syntax (e.g. !skos:exactMatch) or additional predicates like ‘example:notExactMatch’. The former solution (!skos:exactMatch) is a violation of the ‘simplicity’ requirement because it introduces the need to handle special syntax on the user side. The latter solution would have led to a potential doubling of all predicates—which could have led to a combinatorial explosion if it had to capture additional modifiers, such as ‘direct’ or ‘inverse’. The main limitation, and risk, of our chosen approach is that users that consume SSSOM may simply believe that the mappings they consume do not have a predicate modifier (because they never had in the past), and therefore not notice that they suddenly consume ‘negative’ or otherwise modified mappings. We decided that this risk was worth it to keep the model simple and easy to use. A second example where we violate idempotency, i.e. where the addition of additional metadata could change the semantics of pre-existing metadata, is with our ‘preprocessing’ fields—ignoring the ‘preprocessing’ field when interpreting the ‘match_field’ columns could lead to confusing results. For example, ‘Alzheimer 2’ and ‘Alzheimer 3’ are different concepts, but if the nonalphabetical characters were stripped during preprocessing, they would be (misleadingly) matched.

Lack of support for complex mappings. Complex mappings are currently not supported by SSSOM. A complex mapping is a mapping where at least one of the subject or objects of a mapping does not correspond directly to a term, but rather to an expression involving more than one term. Complex mappings are hard to evaluate, but they are receiving interest in the literature (84). During our first workshop, we discussed how complex mappings can be represented, but the majority of the participants were in favor of postponing the introduction of complex mapping to a later stage to protect the simplicity of the current metadata model. We are considering implementing an extension for SSSOM that can capture complex mappings.

## Conclusions and Future Work

Despite the importance of mappings for data integration, standardizing the representation of mappings and mapping rules has not received the same level of care as other ‘semantic artifacts’ such as controlled vocabularies and ontologies. For many use cases, merely providing the subject, object and even predicate of a mapping is not enough, and many mapping sets suffer from nontransparent imprecision, nontransparent inaccuracy, nontransparent incompleteness and unFAIRness, in particular in terms of reusability. Attempts to standardize the representation of mappings are scarce, and generally fall short in three important areas:

1. Insufficient vocabulary for describing metadata in a way that makes imprecision, inaccuracy and incompleteness explicit.
2. Lack of free, open and community-driven collaborative workflows that are designed to evolve the standard continuously in the face of changing requirements and mapping practices.
3. Lack of a standardized tabular representation of a mapping set, which is imperative for facilitating both human curation and use in data science pipelines, and integrates seamlessly with the Linked Data stack.

SSSOM addresses these shortcomings by providing a rich vocabulary for describing mapping metadata, being entirely community-driven with sustainable governance processes in place, and promoting a very simple tabular format for the dissemination of mappings that can be easily integrated in typical data science workflows.

Having a simple standardized format is the main prerequisite for generating high-quality mappings and facilitating their sharing and reuse. The next step, however, is probably even more important, and more difficult: establishing shared best practices for building better mappings. The authors have been working with various groups on improving their manual and automated mapping practices. For example, we worked with OpenTargets (85) to disseminate Mondo-Meddra mappings in SSSOM format. A simple standard for mapping metadata and a simple table format have been instrumental for collaborating with groups such as OpenTargets, IMPC (86), MGI (87) and the Center for Cancer Data Harmonization [CCDH (88)] to build better mapping sets, for example by sharing and editing mapping sets directly through Google Sheets. Developing a metadata standard is usually not enough to improve the quality of data (in our case, mappings) and needs to be accompanied by a set of shared best practices. Analogously to the 5-Star deployment scheme for Linked Data developed by Sir Tim Berners-Lee (https://www.w3.org/2011/gld/wiki/5_Star_Linked_Data), we are developing a 5-Star scheme for rating mappings (89). This 5-star scheme directly evolved out of our experiences working with our collaborators to understand how to best document mappings. It includes considerations such as where and how mapping sets should be published, how they should be licensed and which metadata should be provided.

Our focus in the near future is on developing training materials to help groups to build better mappings and mapping repositories, while continuing to evolve the SSSOM standard and the associated Python toolkit and extending the O×O mapping repository to support SSSOM. We appreciate that not all mapping use cases can be captured by a representation that is deliberately simple, but hope that the medical terminology, database and ontology mapping communities will embrace this more principled approach to disseminating FAIRer and more reusable mapping sets.

## References

[1] Broeder D. , Budroni P., Degl’Innocenti E. et al. (2021) SEMAF: A proposal for a flexible semantic mapping framework. https://zenodo.org/record/4651421#.Yn60VBPMKkg.

[2] Laadhar A. , Abrahão E. and Jonquet C. (2020) Investigating one million XRefs in thirthy ontologies from the OBO world. In: *11th International Conference on Biomedical Ontologies (ICBO)*.

[3] Alignment API . https://moex.gitlabpages.inria.fr/alignapi/ (20 November 2021, date last accessed).

[4] Jackson R. , Matentzoglu N., Overton J.A. et al. (2021) OBO Foundry in 2021: operationalizing open data principles to evaluate ontologies. *Database*, 2021. 10.1093/database/baab069. 10.1093/database/baab069 PMC8546234 34697637

[5] Laadhar A. , Abrahão E. and Jonquet C. (2020) Investigating one million XRefs in thirthy ontologies from the OBO world. In: *ICBO 2020-11th International Conference on Biomedical Ontologies*, Vol. 2807, pp. G.1–12.

[6] Wilkinson M.D. , Dumontier M., Aalbersberg I.J.J. et al. (2016) The FAIR Guiding Principles for scientific data management and stewardship. *Sci. Data*, 3, 160018. 10.1038/sdata.2016.18 PMC4792175 26978244

[7] Miles A. and Bechhofer S. (2009) *SKOS Simple Knowledge Organization System Reference*. W3C Recommendation.

[8] OWL 2 Web Ontology Language Document Overview (Second Edition) . https://www.w3.org/TR/owl2-overview/ (20 November 2021, date last accessed).

[9] Bizer C. , Heath T. and Berners-Lee T. (2011) *Linked Data: The Story so Far. Semantic Services, Interoperability and Web Applications: Emerging Concepts*. IGI Global, pp. 205–227.

[10] SSSOM Specification – SSSOM . http://w3id.org/sssom/spec (18 November 2021, date last accessed).

[11] cmungall-patch-1 · mapping-commons/sssom Github.

[12] CURIE Syntax 1.0 . https://www.w3.org/TR/curie/ (14 November 2021, date last accessed).

[13] McMurry J.A. , Juty N., Blomberg N. et al. (2017) Identifiers for the twenty-first century: How to design, provision, and reuse persistent identifiers to maximize utility and impact of life science data. *PLoS Biol.*, 15, e2001414. 10.1371/journal.pbio.2001414 PMC5490878 28662064

[14] Ciccarese P. , Soiland-Reyes S., Belhajjame K. et al. (2013) PAV ontology: provenance, authoring and versioning. *J. Biomed. Semant.*, 4, 37. 10.1186/2041-1480-4-37 PMC4177195 24267948

[15] Initiative, D.C.M. and Others . (2012) Dublin core metadata element set, version 1.1.

[16] Robinson,A.J.A. and Voronkov,A. (eds). (2001) *Handbook of Automated Reasoning*. Handbook of automated reasoning, North-Holland.

[17] Pesquita C. , Faria D., Falcão A.O. et al. (2009) Semantic similarity in biomedical ontologies. *PLoS Comput. Biol.*, 5, e1000443. 10.1371/journal.pcbi.1000443 PMC2712090 19649320

[18] OBO Metadata Ontology. https://obofoundry.org/ontology/omo (4 December 2021, date last accessed).

[19] SSSOM Specification - SSSOM . https://w3id.org/sssom/spec (7 December 2021, date last accessed).

[20] Haak L.L. , Fenner M., Paglione L. et al. (2012) ORCID: a system to uniquely identify researchers. *Learn. Publ.*, 25, 259–264.

[21] ROR . https://ror.org/ (19 November 2021, date last accessed).

[22] Wikidata:Identifiers . https://www.wikidata.org/wiki/Wikidata:Identifiers (20 November 2021, date last accessed).

[23] Mungall C. LinkML - Linked data Modeling Language - LinkML - Linked data Modeling Language. LinkML - Linked data Modeling Language - LinkML - Linked data Modeling Language. https://linkml.io/ (14 November 2021, date last accessed).

[24] Jimenez-Ruiz E.O. (2020) 0000-0002-9083-4599. Hassanzadeh, O., Efthymiou, V., Chen, J. and Srinivas, K.

[25] Bernard J. (2016) Python data analysis with pandas. In: Bernard J (ed). *Python Recipes Handbook: A Problem-Solution Approach*. Apress, Berkeley, CA, pp. 37–48.

[26] Mungall C. rdf_matcher: swi rdf_matcher. rdf_matcher: swi rdf_matcher; Github.

[27] ecto.sssom.tsv at master · EnvironmentOntology/environmental-exposure-ontology Github.

[28] 1st mapping commons workshop on simple standard for sharing ontology mappings. https://www.wikidata.org/wiki/Q108394519 (4 October 2021, date last accessed).

[29] sssom-py: Python toolkit for SSSOM mapping format Github.

[30] Obographs: basic and advanced OBO Graphs: specification and reference implementation Github.

[31] David J. , Euzenat J., Scharffe F. et al. (2011) The alignment API 4.0. *Semantic Web*, **2**, 3–10.

[32] SSSOM Mapping Format Python Utilities — sssom-py 0.0.1 documentation. https://mapping-commons.github.io/sssom-py/index.html (20 November 2021, date last accessed).

[33] mh_mapping_initiative: repo to organise the mouse-human phenotype mapping initiative and reconcile resources Github.

[34] Vasilevsky N. , Essaid S., Matentzoglu N. et al. (2020) Mondo Disease Ontology: harmonizing disease concepts across the world. In: *CEUR Workshop Proceedings, CEUR-WS*, Vol. 2807.

[35] Amberger J.S. , Bocchini C.A., Scott A.F. et al. (2019) OMIM.org: leveraging knowledge across phenotype-gene relationships. *Nucleic Acids Res.*, 47, D1038–D1043. 30445645 10.1093/nar/gky1151 PMC6323937

[36] Schriml L.M. , Arze C., Nadendla S. et al. (2012) Disease Ontology: a backbone for disease semantic integration. *Nucleic Acids Res.*, 40, D940–6. 22080554 10.1093/nar/gkr972 PMC3245088

[37] Rath A. , Olry A., Dhombres F. et al. (2012) Representation of rare diseases in health information systems: the Orphanet approach to serve a wide range of end users. *Hum. Mutat.*, 33, 803–808. 22422702 10.1002/humu.22078

[38] de Coronado S. , Wright L.W., Fragoso G. et al. (2009) The NCI Thesaurus quality assurance life cycle. *J. Biomed. Inform.*, 42, 530–539. 19475726 10.1016/j.jbi.2009.01.003

[39] World Health Organization . (2019) International Statistical Classification of Diseases and Related Health Problems. (11th ed, ICD-11).

[40] Mungall C.J. , Koehler S., Robinson P. et al. (2019) k-BOOM: A Bayesian approach to ontology structure inference, with applications in disease ontology construction. k-BOOM: A Bayesian approach to ontology structure inference, with applications in disease ontology construction. *bioRxiv*, 2019, 048843.

[41] boomer: Bayesian OWL ontology merging Github.

[42] Jupp S. , Liener T., Sarntivijai S. et al. (2017) OxO-A gravy of ontology mapping extracts. ICBO.

[43] Ontology Xref Service . https://www.ebi.ac.uk/spot/oxo (16 November 2021, date last accessed).

[44] Eloe-Fadrosh E.A. , Ahmed F., Anubhav A. et al. (2021) The National Microbiome Data Collaborative Data Portal: an integrated multi-omics microbiome data resource. *Nucleic Acids Res.*, **50**, D828–D836.

[45] Yilmaz P. , Kottmann R., Field D. et al. (2011) Minimum information about a marker gene sequence (MIMARKS) and minimum information about any (x) sequence (MIxS) specifications. *Nat. Biotechnol.*, 29, 415–420. 21552244 10.1038/nbt.1823 PMC3367316

[46] Buttigieg P.L. , Pafilis E., Lewis S.E. et al. (2016) The environment ontology in 2016: bridging domains with increased scope, semantic density, and interoperation. *J. Biomed. Semant.*, 7, 57. 10.1186/s13326-016-0097-6 PMC5035502 27664130

[47] Leitner F. , Carazo J.M., Bischof J. et al. (2021) EOSC-Life Report on the work of the initial demonstrators. https://pub.uni-bielefeld.de/record/2960900.

[48] Euzenat J. Meilicke C. Stuckenschmidt H. et al. (2011) Ontology alignment evaluation initiative: six years of experience. In: Spaccapietra S (ed). *Journal on Data Semantics XV*. Springer Berlin Heidelberg, Berlin, Heidelberg, pp. 158–192.

[49] Nikooie Pour M.A. , Algergawy A., Amini R. et al. (2020) Results of the ontology alignment evaluation initiative 2020. In:*Proceedings of the 15th International Workshop on Ontology Matching (OM 2020), 2788*, pp. 92–138.

[50] Jiménez-Ruiz E. Cuenca Grau B. Horrocks I. et al. (2009) Ontology Integration Using Mappings: Towards Getting the Right Logical Consequences. In: Aroyo L, Traverso P, Ciravegna F, Cimiano P, Heath T, Hyvönen E, Mizoguchi R, Oren E, Sabou M, Simperl E (eds). The Semantic Web: Research and Applications. Springer Berlin Heidelberg. Heidelberg, Germany, pp. 173–187.

[51] Faria D. , Pesquita C., Santos E. et al. (2013) The agreementmakerlight ontology matching system. In: *On the Move to Meaningful Internet Systems: OTM 2013 Conferences*, Springer Berlin Heidelberg, pp. 527–541.

[52] Jiménez-Ruiz E. and Cuenca Grau B. (2011) *LogMap: Logic-Based and Scalable Ontology Matching. The Semantic Web – ISWC 2011*. Springer Berlin Heidelberg, pp. 273–288.

[53] The Alignment API 4.0 . http://www.semantic-web-journal.net/content/new-submission-alignment-api-40 (20 November 2021, date last accessed).

[54] Hertling S. , Portisch J. and Paulheim H. (2019) Melt-matching evaluation toolkit. In: *International Conference On Semantic Systems*, Springer, Cham, pp. 231–245.

[55] Alexander K. , Cyganiak R., Hausenblas M. et al. (2011) *Describing Linked Datasets with the VoID Vocabulary*.

[56] Gray A.J.G. , Baran J., Marshall M.S. et al. (2015) *Dataset Descriptions: HCLS Community Profile*.

[57] Gray A.J.G. , Brenninkmeijer C., Evelo C. et al. (2013) Dataset descriptions for the open pharmacological space. Working draft, Open PHACTS (September 2012). http://www.openphacts.org/specs/2013/WD-datadesc-20130912 (20 November 2021, date last accessed).

[58] van Iersel M.P. , Pico A.R., Kelder T. et al. (2010) The BridgeDb framework: standardized access to gene, protein and metabolite identifier mapping services. *BMC Bioinform.*, 11, 5. 10.1186/1471-2105-11-5 PMC2824678 20047655

[59] Gray A.J.G. , Groth P., Loizou A. et al. (2014) Applying linked data approaches to pharmacology: Architectural decisions and implementation. *Semant. Web*, 5, 101–113.

[60] Mossakowski T. Codescu M. Neuhaus F. et al. (2015) The distributed ontology, modeling and specification language – DOL. In: Koslow A, Buchsbaum A (eds). *The Road to Universal Logic: Festschrift for the 50th Birthday of Jean-Yves Béziau Volume II*. Springer International Publishing, Cham, pp. 489–520.

[61] Lamacki W.F. (2014) OMG. *CDS Rev.*, 107, 52. 24830113

[62] Common Terminology Services 2^TM^ (CTS2^TM^). https://www.omg.org/cts2/ (23 November 2021, date last accessed).

[63] SNOMED CT Release File Specifications - Release File Specification - SNOMED Confluence . https://confluence.ihtsdotools.org/display/DOCRELFMT/SNOMED+CT+Release+File+Specifications (20 November 2021, date last accessed).

[64] Bodenreider O. (2004) The Unified Medical Language System (UMLS): integrating biomedical terminology. *Nucleic Acids Res.*, 32, D267–70. 14681409 10.1093/nar/gkh061 PMC308795

[65] Metathesaurus - Mapping Projects . (2009).

[66] Nguyen V. , Yip H.Y. and Bodenreider O. (2021) Biomedical vocabulary alignment at scale in the UMLS metathesaurus. In: *Proceedings of the Web Conference 2021, WWW ’21, Association for Computing Machinery*, New York, NY, USA, pp. 2672–2683. 10.1145/3442381.3450128 PMC8434895 34514472

[67] UMLS Reference Manual. Bethesda (MD): National Library of Medicine (US) ; (2009) Sep. Table 9. [Mappings (File = MRMAP.RRF)]. https://www.ncbi.nlm.nih.gov/books/NBK9685/table/ch03.T.mappings_file_mrmap_rrf/.

[68] Smith B. , Ashburner M., Rosse C. et al. (2007) The OBO Foundry: coordinated evolution of ontologies to support biomedical data integration. *Nat. Biotechnol.*, 25, 1251–1255. 17989687 10.1038/nbt1346 PMC2814061

[69] Piñero J. , Ramírez-Anguita J.M., Saüch-Pitarch J. et al. (2020) The DisGeNET knowledge platform for disease genomics: 2019 update. *Nucleic Acids Res.*, 48, D845–D855. 31680165 10.1093/nar/gkz1021 PMC7145631

[70] Vos R.A. , Katayama T., Mishima H. et al. (2020) BioHackathon 2015: Semantics of data for life sciences and reproducible research. *F1000Res*, 9, 136. 10.12688/f1000research.18236.1 PMC7141167 32308977

[71] Jeffery K. , Wittenburg P., Lannom L. et al. (2021) Not ready for convergence in data infrastructures. *Data Intell.*, 3, 116–135.

[72] Callahan T.J. , Wyrwa J.M., Vasilevsky N.A. et al. (2020) OMOP2OBO. https://zenodo.org/record/3902767.

[73] Callahan T.J. OMOP2OBO Wiki. OMOP2OBO Wiki; Github.

[74] Noy N.F. , Griffith N. and Musen M.A. (2008) *Collecting Community-Based Mappings in an Ontology Repository. The Semantic Web - ISWC 2008*. Springer Berlin Heidelberg, pp. 371–386.

[75] BioPortal Mappings - NCBO Wiki . https://www.bioontology.org/wiki/BioPortal_Mappings (14 November 2021, date last accessed).

[76] Ghazvinian A. , Noy N.F. and Musen M.A. (2009) Creating mappings for ontologies in biomedicine: simple methods work. *AMIA Annu. Symp. Proc.*, 2009, 198–202. 20351849 PMC2815474

[77] Hoyt C.T. , Hoyt A. and Gyori B.M. (2021) Biomappings: biopragmatics/biomappings. https://zenodo.org/record/5564589.

[78] Gyori B.M. , Hoyt C.T. and Steppi A. (2021) Gilda: biomedical entity text normalization with machine-learned disambiguation as a service. *bioRxiv*, 2021. https://www.biorxiv.org/content/10.1101/2021.09.10.459803v1. 10.1093/bioadv/vbac034 PMC9710686 36699362

[79] Ontologies Guidelines for Best Practice - Public - Confluence . https://pistoiaalliance.atlassian.net/wiki/spaces/PUB/pages/43089942/Ontologies+Guidelines+for+Best+Practice (24 November 2021, date last accessed).

[80] Requirements for an Ontologies Mapping Tool . https://pistoiaalliance.atlassian.net/wiki/spaces/PUB/pages/46956607/Requirements+for+an+Ontologies+Mapping+Tool (24 November 2021, date last accessed).

[81] Requirements for an Ontologies Mapping service . https://pistoiaalliance.atlassian.net/wiki/spaces/PUB/pages/89554997/Requirements+for+an+Ontologies+Mapping+service (24 November 2021, date last accessed).

[82] Harrow I. , Liener T. and Jimenez-Ruiz E. (2019) Ontology mapping for the laboratory analytics domain. In: *12th International Conference on Semantic Web Applications and Tools for Health Care and Life Sciences, 2849*. Edinburgh, UK, pp. 145–146.

[83] Ontology Xref Service Ontology Lookup Service < EMBL-EBI . Ontology Lookup Service < EMBL-EBI https://www.ebi.ac.uk/ols/index (24 November 2021, date last accessed).

[84] Lima B. , Faria D. and Pesquita C. (2021) Challenges of evaluating complex alignments. http://disi.unitn.it/~pavel/om2021/papers/om2021_LTpaper5.pdf.

[85] Carvalho-Silva D. , Pierleoni A., Pignatelli M. et al. (2019) Open Targets Platform: new developments and updates two years on. *Nucleic Acids Res.*, 47,D1056–D1065. 30462303 10.1093/nar/gky1133 PMC6324073

[86] Muñoz-Fuentes V. , Cacheiro P., Meehan T.F. et al. (2018) The International Mouse Phenotyping Consortium (IMPC): a functional catalogue of the mammalian genome that informs conservation. *Conserv. Genet.*, 19, 995–1005. 30100824 10.1007/s10592-018-1072-9 PMC6061128

[87] Bult C.J. , Blake J.A., Smith C.L. et al. (2019) Mouse Genome Database (MGD) 2019. *Nucleic Acids Res.*, 47, D801–D806. 30407599 10.1093/nar/gky1056 PMC6323923

[88] Center for Cancer Data Harmonization . https://harmonization.datacommons.cancer.gov/ (29 November 2021, date last accessed).

[89] SSSOM Specification - SSSOM . https://mapping-commons.github.io/sssom/spec/ (6 December 2021, date last accessed).

